# Demographics, clinical characteristics, and outcomes of 27,256 hospitalized COVID-19 patients in Kermanshah Province, Iran: a retrospective one-year cohort study

**DOI:** 10.1186/s12879-022-07312-7

**Published:** 2022-03-31

**Authors:** Ezat Hesni, Babak Sayad, Fatemeh Khosravi Shadmani, Farid Najafi, Reza Khodarahmi, Zohreh Rahimi, Arezoo Bozorgomid, Nazanin Sayad

**Affiliations:** 1 Department of Infectious Diseases, Faculty of Medicine, Guilan University of Medical Sciences, Rasht, Iran; 2 Infectious Diseases Research Center, Health Institute, Kermanshah University of Medical Sciences, Kermanshah, Iran; 3 Research Center for Environmental Determinants of Health, Health Institute, Kermanshah University of Medical Sciences, Kermanshah, Iran; 4 Medical Biology Research Center, Health Technology Institute, Kermanshah University of Medical Sciences, Kermanshah, Iran; 5 Department of Clinical Biochemistry, Medical School, Kermanshah University of Medical Sciences, Kermanshah, Iran

**Keywords:** COVID-19, SARS-CoV-2, Prevalence, Risk factors, Mortality, Kermanshah, Iran

## Abstract

**Background:**

Since the first official report of SARS-CoV-2 infection in Iran on 19 February 2020, our country has been one of the worst affected countries by the COVID-19 epidemic in the Middle East. In addition to demographic and clinical characteristics, the number of hospitalized cases and deaths is an important factor for evidence-based decision-making and disease control and preparing the healthcare system to face the future challenges of COVID-19. Therefore, this cohort study was conducted to determine the demographics, clinical characteristics, and outcomes of hospitalized COVID-19 patients in Kermanshah Province, west of Iran.

**Methods:**

This multicenter retrospective cohort study included all suspected, probable, and confirmed cases of COVID-19 hospitalized in Kermanshah Province, Iran during the first year of the COVID-19 pandemic. Demographics, clinical characteristics, outcomes and other additional information of hospitalized patients were collected from the COVID-19 database of the Medical Care Monitoring Center (MCMC) of Kermanshah Province.

**Results:**

Kermanshah Province experienced three waves of COVID-19 infection considering the hospitalization and mortality rates between February 20, 2020 and February 19, 2021. A total of 27,256 patients were included in the study: 5203 (19.09%) subjects were suspected, 9136(33.52%) were probable, and 12,917 (47.39%) were confirmed COVID-19 cases. The mean age of the patients was 53.34 ± 22.74 years and 14,648 (53.74%) were male. The median length of hospital stay among COVID-19 survivors and non-survivors patients were 4 (interquartile range [IQR] 1–6) and 4 (IQR 1–8) days, respectively. Among patients with COVID-19, 2646 (9.71%) died during hospitalization. A multivariable logistic regression revealed that odds of death among patients ≥ 85 years was significantly greater than among patients < 15 years (adjusted odds ratio [aOR] 4.79, 95% confidence interval [CI] = 3.43–6.71, p≤ 0.001). Patients with one (aOR 1.38, 95% CI 1.21–1.59, p = 0.04), two (aOR 1.56, 95% CI 1.27–1.92, p = 0.001) or more (aOR 1.50, 95% CI 1.04–2.17, p = 0.03) comorbidities had higher odds of in-hospital death compared to those without comorbidities. The male sex (aOR 1.20, 95% CI 1.07- 1.35, p = 0.002), ICU admission (aOR 4.35, 95% CI 3.80–4.97, p < 0.001), intubation (aOR 11.09, 95% CI 9.58–12.84, p < 0.001), respiratory distress (aOR 1.40, 95% CI 1.22–1.61, p < 0.001), loss of consciousness (aOR 1.81, 95% CI 1.45–2.25, p < 0.001), anorexia (aOR 1.36, 95% CI 1.09–1.70, p = 0.006) and peripheral oxygen saturation (SpO2) < 93(aOR 2.72, 95% CI 2.34–3.16, p < 0.001) on admission were associated with increased risk of death in patients with SARS-CoV-2 infection. Having cough (aOR 0.82, 95% CI 0.72–0.93, p = 0.003) and headache (aOR 0.70, 95% CI 0.50–0.97, p = 0.03) decreased the odds of death.

**Conclusion:**

The mortality rate of the patients admitted to the general wards and ICU can be a guide for allocating resources and making appropriate plans to provide better medical interventions during the COVID-19 pandemic. Several risk factors are associated with the in-hospital mortality of COVID-19, including advanced age, male sex, ICU admission, intubation, having comorbidity, SpO2 < 93, respiratory distress, loss of consciousness, headache, anorexia, and cough. These risk factors could help clinicians identify patients at high risk for death.

**Supplementary Information:**

The online version contains supplementary material available at 10.1186/s12879-022-07312-7.

## Background

At the end of 2019, severe acute respiratory syndrome coronavirus 2 (SARS-CoV-2) was detected in Wuhan, the capital of Hubei Province, Central China [1]. The disease rapidly spread across China and the world and became a major health concern on a global scale. It has a broad spectrum of clinical manifestations from flu-like symptoms such as fever, cough, anosmia, headache, myalgia and gastrointestinal symptoms to severe acute respiratory failure, renal failure, and need for hemodynamic support [2]. While most the infected patients have mild symptoms and some may even be asymptomatic, approximately 3–5% of the patients will require hospitalization [3]. However, the exact rates differ per country. Microbial and fungal co-infection in COVID-19 patients also remain a life-threatening condition [4].

The first two cases of COVID-19 in Iran were reported in Qom Province on February 19 [5]. Afterward, a wave of the pandemic outbreak hit the country. During the earliest months of the COVID-19 outbreak, the adjusted seroprevalence was 17.1% (95% CI 14.6–19.5) ranging from 72.6% (95% CI 53.9–92.8) to 1.7% (95% CI 0.0–6.0) in a population-based seroprevalence study using sera samples obtained from 3530 individuals from the general population from 18 cities from 17 provinces in country[6]. Despite the economic crisis in Iran, it has taken important steps to combat COVID-19 since the earliest days of the pandemic [7, 8]. Nevertheless, the increasing number of patients with COVID-19 has increased the demand for hospitalization and admission to the intensive care unit (ICUs). The emergency preparedness in terms of hospital beds, skilled health workers, reserves of personal protective equipment and consumables and pharmaceuticals differs markedly between countries, which can lead to differences in mortality rates.

Several studies have investigated the risks factors and mortality rates of SARS-CoV-2 infection [9–12], but there is a lack of comprehensive data from Iran as one of the important epicenters of the COVID-19 pandemic in the Middle East. These data allow authorities to assess the risk factors, strategies, and infection control practices. Thus, this observational study was conducted to describe the demographics, clinical characteristics and outcomes of COVID-19 patients admitted to the hospitals of Kermanshah Province, West of Iran during a one -year period.

## Methods

### Setting and study design

Kermanshah Province is one of the 31 provinces of Iran located in the west of the country bordering Iraq. According to the 2017 census, it is the 13^th^ most populated province in Iran with a population of 1,952,434. Kermanshah Province comprises 14 counties including Kermanshah, Dalahu, Gilan-e Gharb, Harsin, Eslamabad-e Gharb, Javanrud, Kangavar, Paveh, Qasr-e Shirin, Ravansar, Sahneh, Sarpol-e Zahab, Salas-e Babajani, and Sonqor. Kermanshah Province, as the center of medical treatment in the west of the country, covers many patients from adjacent provinces as well as Iraq every year.

In this retrospective, multicenter cohort study, we enrolled all patients who were hospitalized with SARS-CoV-2 infection to 2 treatment centers and 25 hospitals (8 university hospitals, 10 governmental hospitals, 2 private hospitals, 3 military hospitals, and 2 hospitals affiliated to other organizations) in Kermanshah Province, Iran, between February 20, 2020 and February 19, 2021. All hospitals were supervised by KUMS (Kermanshah University of Medical Sciences) during the COVID-19 pandemic. All hospitalized patients were followed-up until they were either discharged or died.

The inclusion criterion was hospitalization with suspected, probable or confirmed diagnosis of COVID-19 during the study period. It should be noted that only cases with moderate to severe COVID-19 (peripheral oxygen saturation (SpO2) of 93% or less in room air and/or an absolute lymphocyte count of < 1100/μl, blood pressures (BPs) < 90 mmHg or decreased level of consciousness) were admitted to the hospital as per guidelines issued by Iran Ministry of Health and Medical Education (MOHME). The subjects that lacked a positive RT-PCR test, positive computed tomography (CT) findings, and congruent symptoms were excluded from the study. In addition, patients with duplicate records based on name, ID number and gender were excluded.

### Study definitions

According to guidelines issued by the MOHME, COVID-19 patients were classified as suspected, probable, or confirmed cases. Briefly, the suspected COVID-19 cases were defined as those having symptoms such as fever, cough or acute respiratory illness. Probable cases were defined as suspected cases that had chest CT scan showing findings of COVID-19 disease. Confirmed cases were defined as individuals with a positive real-time RT-PCR test or nucleic acid sequencing for the SARS-CoV-2 virus based on nasopharyngyal swab samples with/without typical radiological findings. The history of exposure to SARS-CoV-2 was defined as having any close contacts with a confirmed or probable COVID-19 case in the 14 days prior to onset of symptoms. Re-infection with COVID-19 was defined as a person was infected (got sick) once, recovered, and then later became infected again. The history of exposure to SARS-CoV-2 and re-infection with COVID-19 was assessed by questioning the patient.

### Data sources and collection

At the beginning of the COVID-19 epidemic, Iranian MOHME set up Medical Care Monitoring Center (MCMC) information system for reporting hospitalized COVID-19 patients. All suspected, probable, and confirmed case data in Kermanshah Province were daily recorded on the national registry of COVID-19 database according to Iranian MOHME guideline. In this portal, demographic variables such as age and sex, self-reported history of comorbidity (including hypertension, cardiovascular disease, immunodeficiency, cancer, pulmonary disease, etc.), smoking status, drug addiction, pregnancy, history of exposure to SARS-CoV-2, and previous COVID-19 infection registered for each patient. In addition, this portal included self-reported symptoms including symptom onset to hospital admission (including respiratory distress, fever, cough, muscular pain, anorexia etc.), chest CT scan report, RT-PCR result, intubation, ICU admission and in-hospital outcome (death or discharge). In this multi-centre study, the data of all COVID-19 patients who had visited COVID-19 designated healthcare facilities across the province of Kermanshah were extracted from the KUMS integrated MCMC web-based database The data from all designated hospitals for COVID-19 in province were registered in the portal in the same format and the central office in KUMS had access to all records anytime.

### Statistical analysis

Continuous variables were expressed as means ± standard deviation (SD) or median and interquartile range (IQR). Categorical variables were presented as frequencies and percentages. Univariate and backward stepwise multivariate logistic regression models were performed to estimate the association between clinical and demographic characteristics of COVID-19 patients with in-hospital mortality. Variables from univariate models with p < 0.2 were included in a backward stepwise logistic regression model (comorbidities together are considered in the model). Results from the multivariate logistic model are presented as adjusted OR (aOR) with 95% confidence interval [13] and p-value. All p-values of < 0.05 were considered statistically significant. Statistical analysis was performed using Stata version 14.1 software (Stata Corp, College Station, TX, USA).

## Results

During a 1-year period, 28,996 patients with SARS-Co-V2 infection were admitted to the designated hospitals, of whom 809 patients were excluded due to duplicate records based on the name, ID number, time of admission, hospital, and 931 patients were excluded due to lack of PCR-confirmed SARS-CoV-2, CT scan and congruent symptoms. Finally, 27,256 COVID-19 patients with a mean age of 53.34 ± 22.74 years (median, IQR: 57, 38–70) were included in the study (53.74% male and 46.26% female). Of all patients, 11,002 (40.37%) were in the age group 60–85 years. Table 1 shows the demographic and clinical characteristics of 27,256 patients included in this study.

**Table 1:** Clinical and demographic characteristics for 27,256 cases involved in this study by mortality

Variable	Total N (%) (n = 27,256)	Died N (%) (n = 2646)
Gender		
Male	14,648(53.74)	1533 (57.94)
Female	12,608(46.26)	1113 (42.06)
Age (Years)		
< 15	2122 (7.79)	102 (3.85)
15–45	6635 (24.34)	240 (9.07)
45–60	5893 (21.63)	403 (15.23)
60–85	11,002 (40.37)	1568 (59.26)
> 85	1604 (5.88)	333 (12.59)
Dwelling place		
Provincial capital	13,487(49.48)	1579 (59.67)
Outside provincial capital	12,987(47.65)	1002 (37.87)
Other provinces	782(2.87)	65 (2.46)
History of contact with a case of COVID 19 in the last 14 days	13,826 (50.72)	1377 (52.04)
Re-infection with COVID-19	226 (0.82)	28 (1.54)
Smoking	666 (2.44)	64 (2.42)
Drug addiction	539 (1.97)	53 (2)
Pregnancy	260 (0.95)	1 (0.04)
Presenting symptoms		
Respiratory distress	17,875 (65.58)	1894 (71.53)
Cough	11,276 (41.37)	764 (28.85)
Fever	8417 (30.88)	542 (20.47)
Muscle pain	6987 (25.63)	502 (18.96)
Anorexia	1947 (7.14)	163 (6.24)
Headache	1550 (5.68)	62 (2.38)
Loss of consciousness	1748 (6.41)	426 (16.09)
Nausea	1274 (4.67)	73 (2.79)
Vomiting	219 (0.80)	53 (2.03)
Chest pain	1108 (4.06)	77 (2.96)
Abdominal pain	810 (2.97)	34 (1.30)
Diarrhea	758 (2.78)	29 (1.11)
Dizziness/Vertigo	512 (1.87)	30 (1.15)
Loss of smell	481 (1.76)	22 (0.83)
Loss of taste	413 (1.51)	26 (0.98)
Convulsions	210 (0.77)	10 (0.38)
Paresis	177 (0.64)	13 (0.50)
Palegia	84 (0.30)	11 (0.42)
Dermatitis	78 (0.28)	7 (0.27)
Type of comorbidity		
Hypertension	3448 (12.65)	499 (18.92)
Cardiovascular disease	2077 (7.62)	284 (10.77)
Diabetes	2008 (7.36)	277 (10.50)
Cancer	557 (2.04)	88(3.34)
Chronic kidney disease	479 (1.75)	96 (3.64)
Asthma	310 (1.13)	26 (0.99)
Other chronic lung diseases	493 (1.80)	60 (2.27)
Chronic neurologic diseases	140 (0.51)	23 (0.87)
Chronic blood disease	118 (0.43)	13 (0.59)
Chronic liver disease	96 (0.35)	19 (0.72)
Immunodeficiency	36 (0.13)	2 (0.08)
HIV/AIDS	19 (0.07)	1 (0.04)
Number of comorbidities, n (%)		
1	4968(18.22)	654 (24.79)
2	1699(6.2)	255(9.67)
≥ 3	369(1.35)	58 (2.20)
Saturation of O2 < 93	17,799 (65.30)	2194 (82.84)
Case definition		
Confirmed case	12,917(47.39)	1692 (6395)
Probable case	9136 (33.52)	663 (25.06)
Suspected case	5203 (19.09)	291 (11)
Intensive care unit (ICU) admission	5190 (19.04)	1111 (41.99)
Intubation	3163 (11.60)	1004 (37.94)

### Clinical presentation on admission and comorbidities

In total, 5203 (19.09%) subjects were suspected, 9136 (33.52%) were probable, and 12,917 (47.39%) were confirmed COVID-19 cases. The most common symptoms in the time of admission were respiratory respiratory distress (65.58%), cough (41.37%), fever (30.88%), and muscle pain (25.63%). Approximately one-fifth of COVID-19 patients (18.22%) had one comorbidity, and the most common comorbidities were hypertension (12.65%), cardiovascular disease (7.62%), diabetes (7.36%), and cancer (2.04%) (more details in Table 1).

### Geographical distribution of hospitalization and mortality

**Fig. 1:**
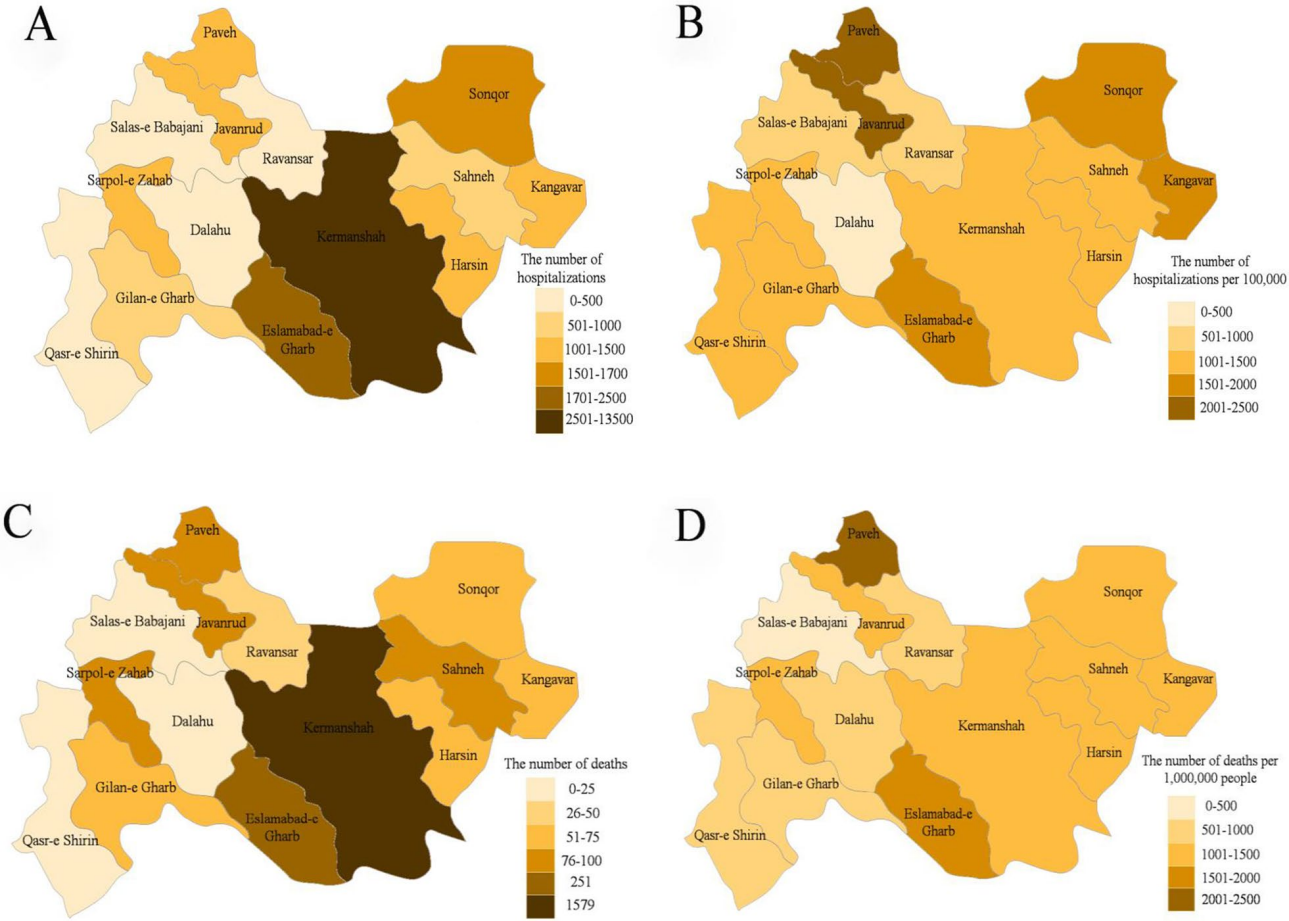
Distribution of hospitalization and death cases from COVID-19 at the county scale in Kermanshah province from February 20, 2020-February 19, 2021 (782 non-resident patients in Kermanshah Province were excluded from the map): **A**: the number of hospitalization, **B**: the number of hospitalization per 100,000 population by county, **C**: the number of deaths, **D**: the number of deaths per 1,000,000 population by county

Figure 1 shows map the distribution of hospitalized COVID-19 patients based on county of residence. According to the results, the frequency of hospitalized COVID-19 patients in Kermanshah Province was 1312 in 100,000 population, ranging from 425 per 100,000 to 2472 per 100,000 in Dalahu and Paveh counties, respectively (Fig. 1B and Additional file 1: Table S1). During the 1-year period, the frequency of death among patients hospitalized with COVID-19 in Kermanshah Province was 1280 per 1,000,000 population. The lowest and highest the frequency of death among patients hospitalized with COVID-19 was 388 per 1,000,000 and 2472 per 1,000,000 population in Salas-e Babajani and Paveh counties, respectively (Fig. 1D and Additional file 1: Table S1).

The highest frequency of hospital admissions and deaths was seen in three referral hospitals: Farabi (14.11% of hospital admissions and 11.88% of deaths), Imam Reza (12.38% of hospital admissions and 6.93% of deaths) and Golestan (11.73% of hospital admissions and 19.45% of deaths). The highest frequency of ICU admission was seen in Imam Ali Hospital (416 cases, 58.84%). In addition, the highest frequency of ICU deaths was seen in Shahid Chamran hospital in Kangavar county (46 cases, 71.88%) (more details in Additional file 1: Table S2).

### Clinical outcomes

Of the 27,256 hospitalized COVID-19 patients, 2646 died (9.71%), including 1533(57.94%) female and 1113 (42.06%) male patients. The deceased had a mean age of 64.97 ± 19.97 years compared to those who hospitalized (53.34 ± 22.74) (not shown in the table). In-hospital mortality was 3.85%, 9.07%, 15.23%, 59.26% and 12.59% in the age group < 15 years, 15–45 years, 45–60 years, 60–85 years, and > 85 years, respectively. The ICU admission rate was 19.04% (5190 patients) with a mortality rate of 41.99%. In addition, 11.60% (3163 patients) required invasive mechanical ventilation of whom 37.94% died. Other findings are shown in Table 1.

The median and mean length of hospital stay among COVID-19 survivors patients was 4 (interquartile range [IQR] 1–6) and 4.86 (SD: 5.11) days, respectively. The median and mean length of hospital stay among COVID-19 survivors patients was 4 (IQR: 1–8) and 6.1 (SD: 6.71) days, respectively (Additional file 1: Table S3).

### Epidemiological curve

**Fig. 2:**
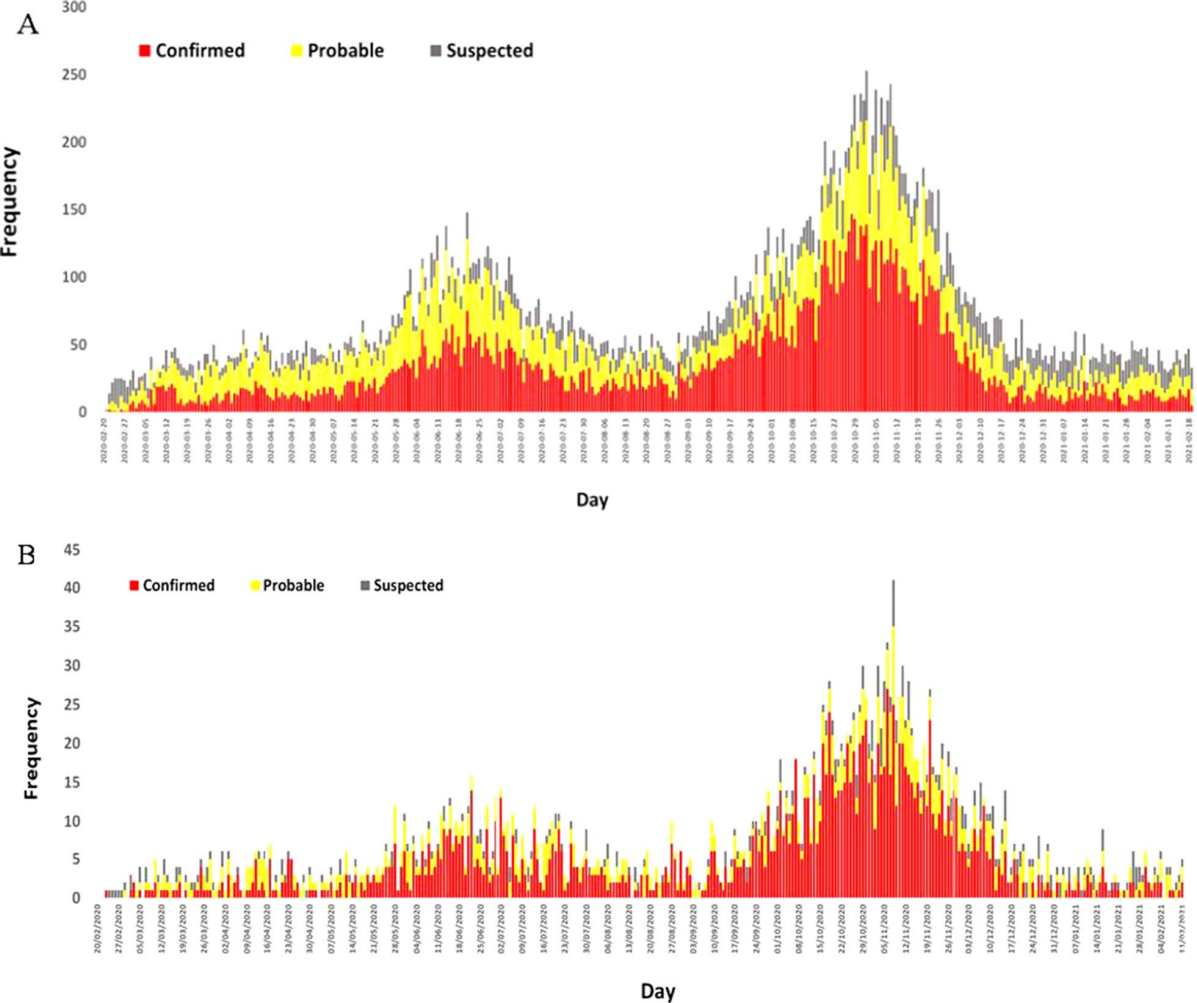
Number of SARS-CoV-2 hospitalized **A** and death **B** cases from 20 February 2020 to 19 February 2021 in Kermanshah Province, Iran

Kermanshah Province experienced three waves of COVID-19 infection considering the hospitalization and mortality rates in one year. The daily distribution of hospital admissions and in-hospital mortality is shown in Fig. 2. The first wave was between early March and early May 2020. The largest number of daily admissions and in-hospital mortality was 189 and 6 cases in the first wave, respectively. The second and third waves hit dramatically in late May and early September followed by a progressive decrease with very few patients admitted in late August and late December. The largest number of daily admissions and in-hospital mortality was 488 and 16 cases in the second wave and 1244 and 38 cases in the third wave, respectively.

### Predictors of in-hospital all-cause mortality

A multivariable logistic regression revealed that odds of death among patients ≥ 85 years was significantly greater than among patients < 15 years (adjusted odds ratio [aOR] 4.79, 95% confidence interval [CI] = 3.43–6.71, p < 0.001). Male sex was also associated with increased odds of in-hospital mortality (aOR 1.20, 95% CI 1.07- 1.35, p = 0.002). The patients with SpO2 ≤ 93% using a pulse oximeter had 2.7(95% CI 2.34–3.16, p < 0.001) times higher odds of mortality compared to those with SpO2 > 93% in the multivariable model. Patients with one (aOR 1.38, 95% CI 1.21–1.59, p = 0.04), two (aOR 1.56, 95% CI 1.27–1.92, p = 0.001) or more (aOR 1.50, 95% CI 1.04–2.17, p = 0.03) comorbidities had higher odds of in-hospital death compared to those without comorbidities. Multivariable regression showed increased odds of mortality rate with respiratory distress (aOR 1.40, 95% CI 1.22–1.61, p < 0.001), loss of consciousness (aOR 1.81, 95% CI 1.45–2.25, p < 0.001), and anorexia (aOR 1.36, 95% CI 1.09–1.70, p = 0.006) on admission. Having cough (aOR 0.82, 95% CI 0.72–0.93, p = 0.003) and headache (aOR 0.70, 95% CI 0.50–0.97, p = 0.03) decreased the odds of death. There was a statistically significant association between COVID-19 death and ICU admission (aOR 4.35, 95% CI 3.80–4.97, p < 0.001) and intubation (aOR 11.09, 95% CI 9.58–12.84, p < 0.001). The risk factors for in-hospital are summarized in Table 2.

**Table 2:** Related factors associated with death in hospitalized COVID-19 patients in Kermanshah Province, Iran

Variable	Category	Total number (%)	Crude OR	P value	Adjusted OR (95% CI)*	P value
Sex	Male	14,648(53.74)	1.20 (1.11–1.31)	< 0.001	1.20 (1.07–1.35)	0.002
	Female	12,608 (46.26)	1		1	
Age Group	< 15	2122 (7.79)	1		1	
	15–45	6635 (24.34)	0.74 (0.58–0.94)	0.01	0.81 (0.58–1.13)	0.23
	45–60	5893 (21.62)	1.45 (1.16–1.81)	< 0.001	1.43 (1.04–1.96)	0.02
	60–85	11,002 (40.37)	3.29 (2.68–4.05)	< 0.001	2.88 (2.14–3.87)	< 0.001
	≥ 85	1604 (5.88)	5.18 (4.11–6.54)	< 0.001	4.79 (3.43–6.71)	< 0.001
Dwelling place	Outside provincial capital and other provinces	13,769 (50.52)	0.79 (0.58–0.68	< 0.001	1.14 (1.01–1.29)	0.03
	Provincial capital	13,487 (49.48)	1		1	
History of contact with a case of COVID 19	Yes	12,449 (45.67)	1.32 (1.22–1.44)	< 0.001	0.89 (0.79–1.01)	0.08
	No	14,807 (54.33)	1		1	
Length of stay in hospital (Day)			1.03 (1.02–1.03)	< 0.001	1.00 (0.99–1.00)	0.48
Intubation	Yes	2159 (7.92)	12.40 (11.24–13.68)	< 0.001	11.09 (9.58–12.84)	< 0.001
	No	25,097 (92.08)	1		1	
Intensive care unit (ICU) admission	Yes	4079 (14.97)	5.27 (4.83–5.75)	< 0.001	4.35 (3.80–4.97)	< 0.001
	No	23,177 (85.03)	1		1	
Number of comorbidities	1	4314 (18.31)	1.58 (1.44–1.74)	< 0.001	1.38 (1.21–1.59)	0.04
	2	1444 (6.13)	1.88 (1.63–2.17)	< 0.001	1.56 (1.27–1.92)	0.001
	≥ 3	374 (1.59)	1.86 (1.43–2.40)	< 0.001	1.50 (1.04–2.17)	0.03
	No	17,430 (73.98)	1		1	
Body temperature (C°)			0.84 (0.79–0.90)	< 0.001	0.88 (0.83–0.94)	< 0.001
Hospital	Referral hospitals	10,258 (37.64)	1.81 (1.67–1.97)	< 0.001	1.75 (1.54–2.00)	< 0.001
	Other hospitals	16,998 (62.36)	1		1	
Case definition	Confirmed case	12,917(47.39)	2.54 (2.23–2.89)	< 0.001	2.17 (1.78–2.65)	< 0.001
	Probable case	9136 (33.52)	1.35 (1.14–1.52)	< 0.001	1.19 (0.95–1.48)	0.11
	Suspected case	5203 (19.09)	1		1	
Saturation of O2	< 93	15,605 (57.25)	4.03 (3.63–4.47)	< 0.001	2.72 (2.34–3.16)	< 0.001
	≥ 93	11,651 (42.750	1		1	
Symptoms	Fever	7875 (28.89)	0.60 (0.59–0.66)	< 0.001	1.07 (0.92–1.24)	0.35
	Cough	10,512 (38.57)	0.61 (0.56–0.67)	< 0.001	0.82 (0.72–0.93)	0.003
	Respiratory distress	15,981 (58.63)	1.87 (1.71–2.04)	< 0.001	1.40 (1.22–1.61)	< 0.001
	Loss of consciousness	1322 (4.55)	5.07 (4.48–5.73)	< 0.001	1.81 (1.45–2.25)	< 0.001
	Convulsions	200 (0.73)	0.48 (0.25–0.92)	0.02	0.77 (0.33–1.83)	0.56
	Abdominal pain	776 (2.97)	0.40 (0.28–0.57)	< 0.001	0.75 (0.46–1.24)	0.27
	Vomiting	1166 (4.46)	0.41 (0.31–0.55)	< 0.001	0.87 (0.60–1.26)	0.49
	Anorexia	1784 (6.83)	0.89 (0.76–1.06)	0.19	1.36 (1.09–1.70)	0.006
	Headache	1488 (5.76)	0.37 (0.28–0.48)	< 0.001	0.70 (0.50–0.97)	0.03
	Muscle pain	6485 (23.79)	0.72 (0.65–1.80)	0.21	–	–
	Loss of taste	387 (1.42)	0.66 (0.44–1.99)	0.46	–	–
	Nausea	1201 (4.60)	0.57 (0.44–1.41)	0.39	–	–
	Dizziness/vertigo	482 (1.87)	0.58 (0.40–1.85)	0.51	–	–
	Paresis	164 (0.63)	0.76 (0.43–1.35)	0.35	–	–
	Plegia	73 (0.28)	1.54 (0.83–3.01)	0.26	–	–
	Dermatitis	71 (0.27)	0.97 (0.44–2.13)	0.95	–	–
	Chest pain	1031 (3.99)	0.71 (0.56–1.90)	0.55	–	–
	Diarrhea	729 (2.79)	0.36 (0.25–1.53)	0.43	–	–

*Odds Ratio for each variable adjusted for other variables in model

*CI* confidence interval, *OR* odds ratio

## Discussion

This study investigated the epidemiological, demographic, and clinical characteristics and outcomes of COVID-19 patients hospitalized in all hospitals and healthcare facilities of Kermanshah Province, west of Iran during the first year of the pandemic. In the present study, 27,256 patients with SARS-Co-V2 infection were admitted to the hospitals. Our results show that the in-hospital mortality rate in the hospitalized patients with COVID-19 was 9.71% (2646 of 27,256). In-hospital mortality rate varied between previous studies conducted in Iran, reporting the earlier mentioned 8.5% in an Ardabil Province cohort[14], 27.62% in a Mashhad city [15], and about 10% in a Tehran Province cohort. In a nationwide study in Iran between 20 February and 20 April 2020, the cumulative risk for in-hospital mortality in 30 days was 24.4% (23,367 of 5693 patients) [16]. In a nationwide study in Brazil including 254,288 hospitalized patients with PCR confirmed COVID-19, in-hospital mortality was 38% (87,515 of 232 036 patients); moreover, 59% (4 002 of 79,687) of the patients were admitted to the ICU, and 80% (36,046 of 45,205) required mechanically ventilated [17]. Mortality among hospitalized COVID-19 patients in USA [18], Oman [19], India [20] is reported 21.4%, 26% and 13.72%, respectively. Although the criteria for hospital admission, case definitions, patient characteristics, etc. may vary among countries, the mortality of COVID-19 patients could be influenced by the mismatch between demand and supply, resource capacity, and lack of trained ICU staff.

In our study, the median (IQR) length of hospital stay was 4 (1–6) and 4 (1–8) day in survivors and non-survivors, respectively, which was shorter than those reported in large cohorts in other countries such as Indonesia [21], Belgian [22] and Germany [23]. The presence of comorbidities, patient age, delayed access to healthcare, and hospital discharge criteria can be responsible for this difference. Iran was one of the countries that experienced the highest number of COVID-19 cases and deaths. Therefore, in order to increase the capacity of the hospitals in response to pandemic, especially at the peak of COVID-19 outbreaks, a number of patients were discharged from hospital with partial recovery and moved to home care.

Our findings are in line with two previous cohort studies, which reported cough was associated with good prognosis in patients hospitalized with COVID-19 [24, 25]. Possible reason could be role of earlier health seeking behavior in patients with cough that contributing to more favorable outcomes for patients. Nevertheless, these findings conflict with several other studies which found cough is an adverse predictor of case fatality or severe disease [26]. It is important to note that we didn’t specify the nature of cough, thus these results should be interpreted with caution.

Critical COVID-19 may be associated with acute respiratory distress syndrome (ARDS), liver damage, heart failure, renal failure, shock cardiac and septic shock [27]. Hence, ICU admission plays a dominant role in the care of COVID-19 patients and reducing the mortality. In a Chinese study, mortality rate of outside ICUs in patients with COVID-19 was 30% [28], and it was 13% in patients hospitalized with COVID-19 in Brazil [13]. Previous studies from Iran have reported a range of 46% to 88% [14, 15, 29]. The results of our study showed that mortality rate outside of ICU (60%) were higher compared to the mentioned studies. Although, the increased number of critically ill patients during COVID-19 peaks increases the demand for critical care, health authorities need to increase ICU beds, particularly in referral hospitals, to avoid delays in admission and provide mechanical ventilators in abundance to treat the patients who present with respiratory distress.

Advanced age is an independent risk factor for COVID-19 mortality [30]. A recent meta-analysis found that adults above 65 years were five times more likely to become critical or die [31]. In the present study, adjusted in-hospital mortality estimates associated with COVID-19 were the lowest in the age group 0–15 years and increased almost linearly with age from 2.88- fold odds (95% CI 2.14–3.87, P < 0.001) in patients 60–85 years to 4.79- fold odds (95% CI 3.43–71.6, P < 0.001) in those ≥ 85 years. Some studies showed that advanced age was associated with a compromised immune system and an increase in chance of co-morbidities resulting in a higher COVID mortality, similar to SARS and MERS [32, 33]. In the present study, more than half of the hospitalized cases were male patients (53.74%), which was consistent with other studies [34, 35]. It was also found that male gender significantly increased mortality with an adjusted odds ratio of 1.20 (95% CI: 1.07–1.35). This finding was also consistent with other studies [36, 37]. The lower incidence of SARS-CoV-2 among women may be related to the physiology of human (X chromosome and sex hormones) which have been reported to play a major role in innate and adaptive immunity [38].

Interestingly, the in-hospital mortality rate of COVID-19 in pregnant women was very low in our study (1 case per 259 pregnant women). A multicenter cohort study of COVID-19 in hospitalized pregnant women from USA evaluated 44(69%) pregnant women with severe disease and 20(31%) with critical disease [39]. The authors concluded that pregnant women might experience similar, or even lower, rates of severe or critical COVID-19 than non-pregnant patients. They did not report any deaths. Similarly, a systematic review found that the mortality of the pregnant women with COVID-19 was lower than that of overall COVID-19 patients [40]. In addition, a universal screening study in Mexico showed no difference in perinatal outcomes between SARS-CoV-2 positive and negative pregnant women, except for preeclampsia [41]. This is possible that younger pregnant women have a lower risk of underlying diseases. Overall, little evidence is available on COVID-19 in pregnant women and further research is warranted.

Nowruz, the celebration of Iranian New Year, starts on March 20/21 and is considered the most important holiday in Iran. The outbreak of COVID-19 in Iran coincided with the end of the Persian year which is characterized by a shopping spree and crowded streets. On the other hand, Nowruz holidays are a time for Iranians to make family gatherings, ceremonies, and travels. Thus, Nowruz holidays aggravated the COVID-19 crisis in Iran and the first wave of the epidemic started in the mid-spring [42]. Following the increase in the number of new cases of COVID-19, the government imposed some restrictions such as travel banning, closure of the schools and universities, a ban on Friday prayers and working from home. Considering that the spread of COVID- 19 infection has never been completely under control in Iran, Kermanshah Province experienced other two major peaks in hospitalized and mortality cases with a slight delay compared to some provinces in mid-summer, and mid-autumn. In line with other reports from Iranian health officials, our findings also showed an increase in the total number of hospitalizations and deaths during the third wave compared to the previous waves [32]. The highest daily mortality from COVID-19 in Iran and Kermanshah Province in 2020 was 486 (on 2020–11-16) [43] and 38 cases (on 2020-11-08), respectively. In November and December 2020, the daily number of mortality for COVID-19 exceeded 400 cases during 28 days in Iran [32]. This high rate of mortality could be associated with several factors such as reduced observance of health protocols and social distancing, failure to enforce interventions due to public fatigue and economic problems, increased travel in the late summer, and lack of specific protocols for travel restrictions. It is worth noting that the third peak coincided with the arrival of B.1.1.413 variants of SARS-CoV-2 in the country which is more contagious than the alpha variant [44]. According to the national vaccination document, vaccination against COVID-19 started on February 21, 2021 with priority given to the health professionals, elderly population, and vulnerable and high-risk groups. Nevertheless, a combination of limited vaccine availability and slow vaccination rate leads to a longer epidemic and may even result in new SARS-CoV-2 variants, which can compromise immunity, raise concerns about vaccine effectiveness, and increase the crisis worldwide.

A strength point of this study was its large sample size including confirmed, probable, and suspected COVID-19 cases; therefore, it may provide a comprehensive picture of hospitalized patients with COVID-19 infection. Our study also suffered from several limitations. First, some variables such as the body mass index, treatments administered in the hospital and laboratory findings were not provided, which limited our ability to draw a conclusion on mortality based on the data. Second, the comorbidity data were based on self-reports and it was not possible to verify their validity. Finally, attention should be paid to variations in patient management; however, all hospitals and healthcare facilities for COVID-19 patients in Kermanshah Province were included in the study.

## Conclusions

In summary, about half of patients hospitalized with COVID-19 in Kermanshah Province were over 60 years of age. Of patients in this cohort, 19.04% of the patients were admitted to ICU of whom 41.99% died. Moreover, 11.60% required invasive mechanical ventilation, of whom 37.94% died. Overall, the in-hospital mortality rate was 9.71%. Although patients requiring intubation were more likely to die, the beneficial effects of intubation on patient treatment and management should not be ignored. Male gender, advanced age, comorbidities significantly increased the odds of mortality. Respiratory distress, SpO2 < 93, loss of consciousness, and anorexia were the symptoms most strongly associated with higher odds of mortality, suggesting that they have a high value in clinical assessment of the severity of COVID-19. As expected, due to the admission of patients with more serious and complex medical conditions in referral hospitals, the odds ratio of COVID-19 related mortality was higher in these hospitals compared other hospitals. Nonetheless, this finding requires the immediate attention of health officials to provide better medical services and preventive interventions for off-center healthcare facilities.

## Supplementary Information

**Additional file 1: Table S1.** Frequency of hospital admissions and deaths by county in Kermanshah Province, Iran, during the first year COVID-19 pandemic. **Table S2.** Outcomes for patients hospitalized with COVID-19 based on hospital. **Table S3.** Duration from hospitalization to discharge/ death for patients hospitalized for COVID-19 based on hospital.

## Data Availability

All data generated or analysed during this study are included in this published article.

## Abbreviations

MCMC: Medical care monitoring center
SpO2: Peripheral oxygen saturation
SARS-CoV-2: Severe acute respiratory syndrome coronavirus 2
ICUs: Intensive care unit
KUMS: Kermanshah University of Medical Sciences
BPs: Blood pressures
MOHME: Ministry of Health and Medical Education
CT scan: Computed tomography
MCMC: Medical care monitoring center
