# Supplementary material for: Demographics, clinical characteristics, and outcomes of 27,256 hospitalized COVID-19 patients in Kermanshah Province, Iran: a retrospective one-year cohort study
Source: BMC Infect Dis. 2022 Mar 31;22:319. doi: 10.1186/s12879-022-07312-7 (PMC8969401; doi:10.1186/s12879-022-07312-7)
Supplement: Supplementary file 1 — Additional file 1: Table S1. Frequency of hospital admissions and deaths by county in Kermanshah Province, Iran, during the first year COVID-19 pandemic. Table S2. Outcomes for patients hospitalized with COVID-19 based on hospital. Table S3. Duration from hospitalization to discharge/ death for patients hospitalized for COVID-19 based on hospital. [file 12879_2022_7312_MOESM1_ESM.docx]

Additional file 1

Table S1 Frequency of hospital admissions and deaths by county in Kermanshah Province, Iran, during the first year COVID-19 pandemic

| **County of residence** | **The frequency of hospital admissions, n%** | **The frequency of hospital admissions per 100,000 people** | **The frequency of deaths, n%** | **The frequency of deaths per 1,000,000 people** |
| --- | --- | --- | --- | --- |
| Eslamabad-e Gharb | 2471(9.07) | 1743 | 254(9.60) | 1792 |
| Paveh | 1442(5.29) | 2472 | 88(3.33) | 2472 |
| Salas-e Babajani | 266(0.98) | 737 | 14(0.53) | 388 |
| Javanrud | 1484(5.44) | 2012 | 77(2.91) | 1044 |
| Dalahu | 157(0.58) | 425 | 20(0.76) | 542 |
| Ravansar | 412(1.51) | 842 | 39(1.47) | 797 |
| Sarpol-e Zahab | 1224(4.49) | 1449 | 90(3.40) | 1065 |
| Sonqor | 1619(5.94) | 1938 | 92(3.48) | 1101 |
| Sahneh | 748(2.74) | 1032 | 74(2.80) | 1021 |
| Qasr-e Shirin | 220(0.81) | 1043 | 18(0.68) | 853 |
| Kermanshah | 13487(49.48) | 1123 | 1579(59.67) | 1316 |
| Kangavar | 1230(4.51) | 1602 | 83(3.14) | 1081 |
| Gilan-e Gharb | 644(2.36) | 1128 | 56(2.12) | 981 |
| Harsin | 1070(3.93) | 1431 | 97(3.66) | 1297 |
| Total in Kermanshah Provincce | 26474(97.13) | 1312 | 2581(97.54) | 1280 |
| Other province | 782(2.87) | - | 65(2/46) | - |

**Table S2 Outcomes for patients hospitalized with COVID-19 based on hospital**

|  | **Hospital name** | **Admission to the hospital**  **( ICU and non-ICU)**  **N= 27256** | | **Admission to the ICU**  **N= 749** | |
| --- | --- | --- | --- | --- | --- |
|  |  | **Total number (%)** | **No. of deaths (%)** | **Total number (%)** | **No. of deaths (%)** |
| **Hospitals of provincial capital (Kermanshah)** | Farabi | 3845 (14.11) | 457(11.88) | 351 (9.13) | 150(42.74) |
|  | Imam Reza | 3374 (12.38) | 234(6.93) | 402(11.911) | 49(12.19) |
|  | Golestan | 3197 (11.73) | 622(19.45) | 658 (20.58) | 316(48.02) |
|  | Dr Mohammad Kermanshahi | 1016 (3.73) | 44(4.33) | 282 (27.76) | 35(12.41) |
|  | Shohada | 1008 (3.70) | 164(16.26) | 175 (17.36) | 66(37.71) |
|  | Imam Hossein | 930 (3.41) | 108(11.61) | 302 (32.45) | 61(20.27) |
|  | Imam Khomeini | 724 (2.66) | 101(13.95) | 113 (15.61) | 54(47.79) |
|  | Ayatollah Taleghani | 707 (2.59) | 112(15.84) | 416 (58.84) | 91(21.88) |
|  | Imam Ali | 679 (2.49) | 78(11.48) | 403 (59.35) | 42(10.42) |
|  | Hazrat Abolfazl Military Hospital | 521 (1.91) | 20(3.83) | 61 (11.71) | 14(22.95) |
|  | Hazrat Masumeh | 286(1.05) | 12(4.19) | 84 (29.37) | 10(11.90) |
|  | Motazedi | 121 (0.44) | 2(1.65) | 41 (33.88) | 1(2.44) |
|  | Army Hospital No. 520 | 28 (0.10) | - | 1 (3.57) | - |
|  | Imam Sajad | 2 (0.01) | - | 0 (0.00) | - |
|  | Bistoon | 442 (1.6) | 8(1.80) | 42 (9.50) | 7(16.67) |
|  | **Total** | **16880(61.93)** | **1962(10.02)** | **3331** | **822(26.47)** |
| **Hospitals of outside provincial capital** | Imam Khomeini (Eslamabad-e Gharb) | 2200 (8.07) | 221(10.04) | 121 (5.50) | 27(22.31) |
|  | Hazrat Rasoul (Javanrud) | 1409 (5.17) | 63(4.47) | 11 (0.78) | 3(27.27) |
|  | Imam Khomeini(Sonqor ) | 1345 (4.93) | 68(5.05) | 186 (13.83) | 47(25.27) |
|  | Ghods (Paveh) | 1322 (4.85) | 67(5.06) | 145 (10.97) | 46(31.72) |
|  | Chamran (Kangavar) | 1081 (3.97) | 64(5.92) | 64 (5.92) | 46(71.88) |
|  | Shohada (Sarpol-e Zahab) | 1020 (3.74) | 56(5.49) | 8 (0.78) | 0 |
|  | Shohada (Harsin) | 876 (3.21) | 58(6.62) | 178 (20.32) | 38(21.35) |
|  | Dr Moaven ( Sahneh) | 587 (2.15) | 53(9.02) | 20 (3.41) | 8(40) |
|  | Al Zahra ( Gilan-e Gharb) | 337 (1.38) | 28(7.42) | 13 (3.45) | 0 |
|  | Abolfazl Abbas (Qasr-e Shirin) | 79 (0.29) | 5(6.32) | 3 (3.80) | 0 |
|  | Bed treatment center (Salas-e Babajani) | 79 (0.29) | 1(1.26) | 0 (0.00) | 0 |
|  | Bed treatment center (Dalahu) | 1 (0.0) | - | - | - |
|  | **Total** | **10376(38.07)** | **684(6.6)** | **749** | **229(30.57)** |
| **Total** | | **27256** | **2646**(**9.71**) | **4079 (14.97)** | **1111**(**27.24**) |

**Table S3 Duration from hospitalization to discharge/ death for patients hospitalized for COVID-19 based on hospital**

| **Hospital name** | **Duration from hospitalization to discharge** | | **Duration from hospitalization to death** | |
| --- | --- | --- | --- | --- |
|  | **Mean±SD** | **Median((IQR )** | **Mean±SD** | **Median (IQR )** |
| Imam Khomeini (Kermanshah) | 6.86±7.34 | 5 (2-8) | 9.94±11.07 | 6 (1-13.5) |
| Farabi(Kermanshah) | 6.83±6.06 | 5 (3-8) | 8.49±8.38 | 6 (2-13) |
| Imam hossein(Kermanshah) | 6.86±7.34 | 5 (2-8) | 9.94±11.07 | 6 (1-13.5) |
| Shohada(Kermanshah) | 5.38±4.25 | 4 (2-7) | 4.91±4.55 | 4 (1-7) |
| Imam Ali(Kermanshah) | 5.19±5.41 | 3 (2-6) | 5.64±7.30 | 2 (1-7) |
| Ayatollah Taleghani(Kermanshah) | 5.14± 5.68 | 4 (2-6) | 5.55±5.07 | 4 (1.5- 8) |
| Imam Reza(Kermanshah) | 5.05±6.03 | 3 (1-6) | 4.48±5.50 | 2 (1-5) |
| Golestan(Kermanshah) | 4.89±4.57 | 4 (1-6) | 5.45±5.42 | 3 (1-8) |
| Dr Mohammad Kermanshahi(Kermanshah) | 4.12±4.74 | 3 (1-5) | 2.52±2.83 | 1 (1-3.5) |
| Hazrat Abolfazl Military Hospital(Kermanshah) | 3.84±5.37 | 1 (1-5) | 10.35±11.33 | 5 (1-13.5) |
| Army Hospital No. 520(Kermanshah) | 3.03±2.15 | 2 (1-5) | - | - |
| Hazrat Masumeh (Kermanshah) | 2.81±2.76 | 2 (2-3) | 2.66±4.09 | 1 (1-1) |
| Motazedi(Kermanshah) | 2.75±4.46 | 1 (1-2) | 3.00±2.82 | 3 (1-5) |
| Imam Sajad(Kermanshah) | 1.00 ±0.0 | 1 (1-1) | - | - |
| Bistoon(Kermanshah) | 2.80±4.03 | 1 (1-3) | 2.37±2.38 | 1.5 (1-2.5) |
| Imam Khomeini ( Sonqor ) | 5.38±4.51 | 5 (2-8) | 4.22±4.24 | 3 (1-5) |
| Dr Moaven ( Sahneh) | 4.71±4.59 | 4 (1-6) | 6.92±7.40 | 5 (1-10) |
| Bed treatment center (Salas-e Babajani) | 4.50± 7.23 | 2 (1-4) | 1.00 | 1(1-1) |
| Chamran (Kangavar) | 4.46±3.46 | 4 (2-6) | 7.07±5.46 | 5 (3-15) |
| Imam Khomeini (Eslamabad-e Gharb) | 4.32 ±4.62 | 3 (1-6) | 5.71±6.13 | 4 (1-8) |
| Al Zahra ( Gilan-e Gharb) | 4.03±3.38 | 3 (2-5) | 5.92±6.22 | 3.5 (2-8.5) |
| Shohada (Harsin) | 3.74±3.48 | 3 (1-5) | 3.62±4.60 | 1 (1-4) |
| Ghods (Paveh) | 3.67±3.49 | 3 (1-5) | 6.77±6.17 | 6 (1-10) |
| Abolfazl Abbas (Qasr-e Shirin) | 3.07±3.21 | 1 (1-5) | 7.20±5.67 | 5 (4-11) |
| Hazrat Rasoul (Javanrud) | 3.04±3.74 | 1 (1-4) | 5.25±5.80 | 3 (1-6) |
| Shohada (Sarpol-e Zahab) | 3.04±3.13 | 2 (1-4) | 4.94±4.51 | 4 (1-7) |
| Bed treatment center (Dalahu) | 2.00 | 2 (2-2) | - | - |
| **Total** | **4.86±5.11** | **4 (1-6)** | **6.10±6.71** | **4 (1-8)** |
